# The intervention effect of *Amygdalus mongolica* oil on the metabolomics and intestinal flora in pulmonary fibrosis

**DOI:** 10.3389/fphar.2022.1037563

**Published:** 2022-11-01

**Authors:** Qian Li, Hong-Bing Zhou, Jia-Qi Liu, Wan-Fu Bai, Jia Wang, Zhan-Jun Yang, Min Qiu, Hong Chang, Song-Li Shi

**Affiliations:** ^1^ Department of Pharmacy, Baotou Medical College, Baotou, China; ^2^ Inner Mongolia Maternal and Child Health Care Hospital, Hohhot, China; ^3^ Institute of Bioactive Substance and Function of Mongolian Medicine and Chinese Materia Medica, Baotou Medical College, Baotou, China

**Keywords:** metabolomics, intestinal flora, Amygdalus mongolica oil, pulmonary fibrosis, Amygdalus mongolica

## Abstract

*Amygdalus mongolica* oil is rich in unsaturated fatty acids such as inoleic acid (47.11%) and oleic acid (23.81%). Our research demonstrates that it exerts a protective effect on rat models of pulmonary fibrosis, however, little is known regarding the underlying mechanism of action. This study aimed to characterize the therapeutic mechanism of action of *A. mongolica* oil on bleomycin-induced pulmonary fibrosis in rats. *A. mongolica* oil appears to regulate the levels of potential key serum biomarkers which include tetrahydrobiopterin, L-serine, citrulline and estradiol to participate in folate biosynthesis, glycine, serine and threonine metabolism, arginine biosynthesis and steroid hormone biosynthesis. And it also enriched intestinal microbial abundance, homogeneity and modulated the abundance of *Duncaniell, Desulfovibrio*, Peptococcaceae*_unclassified*, *Dubosiella*, *Tyzzerella*, Lachnospiraceae*_NK4A136_group, Lactobacillus, Clostridiales_unclassified* to exert a protective effect against pulmonary fibrosis. *A. mongolica* oil appears to confer protective effects against pulmonary fibrosis by affecting the level of pulmonary fibrosis metabolites and the abundance of related intestinal flora through multiple targets, as evidenced by our untargeted LC-MS/MS metabonomics evaluation and 16S rDNA sequencing technology.

## 1 Introduction

Pulmonary fibrosis (PF) is a destructive lung disease with a myriad of etiologies and clinicopathologic features. It is characterized by alveolar epithelial cell injury, excessive proliferation of fibroblasts, the accumulation of a large amount of extracellular matrix accompanied by inflammatory damage, destruction of lung tissue structure, and eventually culminates in respiratory failure as a result of impaired gas exchange and lung function (Prasse et al., 2010; Chanda et al., 2019). Common clinical manifestations are cough, chest tightness, asthma, fatigue, and palpitations (Song et al., 2010). The etiology of PF is largely unknown, and the pathogenesis is complex, with multiple signaling pathways intertwined and possibly including chronic inflammatory responses and cytokine actions. During the development of pulmonary fibrosis disease, the damaged alveolar epithelial cells produce and release large amounts of growth factors and cytokines to stimulate inflammatory cells, and eventually leads to excessive proliferation and migration of fibroblasts (Hu et al., 2021a). In Chinese medicine, pulmonary fibrosis belongs to the category of “lung impotence” and “lung paralysis”. The causes of the disease are mostly external evil attack and mismanagement, and the pathogenesis is mostly deficiency of lung and kidney, and mutual stagnation of phlegm and stasis (Wang et al., 2021). Many different diseases may cause PF. Its affects approximately three million people around the world, and its prevalence sharply increases with age (Martinez et al., 2017). At present, Novel Coronavirus Pneumonia is threatening human health in some countries and most patients still have symptoms such as lung inflammation, pulmonary fibrosis, and dyspnea in the prognosis (Zhan et al., 2020). Therefore, there is an urgent need to discover novel therapeutic drugs of treating and improving the prognosis of PF. Current pharmaceutical agents used to treat PF encompass glucocorticoids, immunosuppressants and cytotoxic drugs, all of which are not efficacious in the long-term and cause several adverse effects. In recent years, Chinese medicine has emerged as a potential candidate in protecting against and treating PF (Liu et al., 2019).

Metabonomics studies the response of metabolites to pathological stimuli and drug treatments in the context of complex biological systems. It can be used to predict the level of metabolites in the body and to guide diagnosis as well as intervention (Wu et al., 2019). Metabonomics adopts a “top-down” strategy to derive the functional status of an organism based on its metabolic networks. Understanding how an intervention changes the metabolism of an organism is very much in line with traditional Chinese medicine dogma (Zhang et al., 2012). Therefore, the application of metabonomics in traditional Chinese medicine has important practical significance for understanding the pharmacodynamics, mechanism of action, guiding the treatment course, as well as in researching traditional Chinese medicine germplasm resources and preclinical safety (Lyu et al., 2018).

Intestinal flora possesses a symbiotic relationship with its human host. Given its astonishing complexity, intestinal microbiota is considered an entire metabolic organ on its own that has the ability to influence human health and participate in the occurrence and development of various diseases (Gosalbes et al., 2012). Intestinal flora is closely related to the respiratory tract, with specific flora possessing the ability to affect the pathogenesis of respiratory diseases (Dumas et al., 2018). The interaction between gut microbes and the lungs is called the gut-lung axis (He et al., 2017). Intestinal flora and lungs interact in pathological states, and many pulmonary diseases such as asthma and lung infections are accompanied by gastrointestinal symptoms. However, the lung diseases can also cause changes in the structure and diversity of the intestinal flora of patients (Tan et al., 2020). The specific flora can help the body maintain a stable immune system balance by reducing the inflammatory response and help the lungs fight infection (Shi et al., 2020). The gut microbiome has the potential to become a powerful diagnostic tool, which can either be modified to create personalized treatment or be monitored over time to aid in diagnosing various diseases early. This will have a profound impact on the improvement of human health and the development of precision medicine (Zhou et al., 2020).

The integration of metabonomics and intestinal flora revealed significant interactions between bacteria and host (Clos-Garcia et al., 2020). Today, intestinal microbiome analysis combined with serum metabonomics has been applied to the pathogenesis and treatment of many diseases such as fibromyalgia, colorectal cancer, hyperuricemia and renal fibrosis (Weir et al., 2013; Clos-Garcia et al., 2019; Hu et al., 2020; Wang et al., 2020). The integrated application of multiple-omics has become an important means of comprehensively using systems biology to study drugs and diseases. The characteristics of multi-component, multi-target, multi-level, and multi-metabolic pathways in traditional Chinese medicine are consistent with the overall, systematic, and comprehensive nature in multi-omics. Using intestinal flora and metabonomics technology, it is possible to carry out a comprehensive analysis of outcomes, and to understand the principles and mechanisms of Chinese medicine in depth, which greatly promotes the research of Chinese medicine and is conducive to the modernization of Chinese medicine (Zhu et al., 2008; Liu et al., 2012).

*Amygdalus mongolica* (Maxim.) Ricker belongs to the Subgen. Amygdalus of Rosaceae *Amygdalus*, and is known as Wulian-Buyilesi in Inner Mongolia and is unique to the Gobi Desert (Yue et al., 2020). It is rich in alkaloids, total flavonoids, polysaccharides, fatty acids, amygdalin, vitamin E, protein, and other medicinal and nutritional ingredients (Shi et al., 2013; Su et al., 2013; Zhu et al., 2013; Bai et al., 2015; Zhou et al., 2015; Jia et al., 2018; Yang et al., 2018). The seeds of *A. mongolica* are a substitute for “Yu Liren” (*Pruni semen*) in traditional Chinese medicine, and is thought to enter the lung meridian to relieve cough and asthma, mainly used to treat dry throat, dry cough, bronchitis, yin deficiency constipation, and edema (Zhao, 1995; Liu et al., 2017). We found that petroleum ether extract of *A. mongolica* can effectively fight liver, lung and kidney fibrosis (Chang et al., 2020; Jia et al., 2020; Quan et al., 2020; Chang et al., 2021; Wu et al., 2021). *A. mongolica* is an important woody oil-bearing tree species, with its kernels consisting of more than 50% oil (Zhang et al., 2016a). The oil is rich in mineral elements such as zinc, iron, calcium, as well as 16 different fatty acids (primarily oleic and inoleic acid, which make up 98% of all its fatty acids) (Wang et al., 2012). It meets all the requirements of the national edible oil grade three quality standard and is a substance with high developmental value. The unsaturated fatty acids in *A. mongolica* oil can play a role in repairing fatty liver damage by enhancing the vitality of liver tissue antioxidant enzymes and antioxidants (Zheng et al., 2017). It can also improve the body’s antioxidant capacity and reduce the degree of renal disease (Hao et al., 2020). Preliminary experimental studies have shown that *A. mongolica* oil can significantly reduce the content of collagen Ⅲ (COL-III), collagen Ⅳ (COL-Ⅳ), hyaluronic acid (HA), laminin (LN), interleukin-1β (IL-1β), interleukin-6 (IL-6), and hydroxyproline in lung tissues, regulate the TGF-β1/Smad signaling pathway, reduce extracellular matrix deposition, and inhibit collagen fiber proliferation to reduce alveolar inflammation and PF (Li et al., 2021).

Therefore, our experiment combined metabolomics and intestinal microflora research to more completely characterize the protective mechanism of *A. mongolica* oil on PF rat models. This investigation provides experimental and scientific evidence on the protective effect of *A. mongolica* oil on the lungs, while also providing new ideas for the rational development and utilization of *A. mongolica* oil resources in the pharmaceutical and food industries.

## 2 Materials and methods

### 2.1 Preparation of *A. mongolica* oil

The seeds of *A. mongolica* were collected from the RuoRigong Alxa in Inner Mongolia. The dry mature seeds were identified by Professor Songli Shi of the Inner Mongolia University of Science and Technology Medical College of Baotou. The seeds are dehulled and crushed into granules. Using the solvent extraction method, the seed kernels are wrapped in filter paper and placed in a soxhlet extractor. Petroleum ether (analytical pure, Tianjin Kaitong Chemical Reagent Co., Ltd, China) is then added. The seeds were subjected to the extraction procedure twice (1 time/2 h) in a water bath. The extract was filtered and dried in order to further concentrate it. The concentrated solution was placed in an oven at 105°C to dry for 2 h, and the supernatant was left to cool to obtain the *A. mongolica* oil. The extraction rate was approximately 87.51%. The oil is then stored in a refrigerator at 4°C until later use. Using GC/MS analysis, we report that the oil content of unsaturated fatty acids comprised of inoleic acid (47.11%), oleic acid (23.81%), palmitic acid (21.37%), palmitoleic acid (2.37%), 8,11 -Octadenoic acid (1.65%), and arachidonic acid (1.25%) (Zhao et al., 2016).

### 2.2 Chemicals and reagents

Pentobarbital sodium was purchased from Merck, Ltd (Germany). Bleomycin powder for injection was purchased from Invitrogen (France). ELISA kit (COL-III, COL-IV, HA, LN, IL-6, IL-1β) was purchased from Jiangsu Immunoenzyme Laboratory Co., LTD (China). Methanol, acetonitrile, and formic acid (Chromatographically pure) were purchased from Fisher (America). E. Z.N.A.®Stool DNA Kit (D4015) was purchased from Omega, Inc (America). AMPure XT beads were purchased from Beckman Coulter Genomics (America). A Qubit dsDNA HSA ssay Kit were purchased from Invitrogen (America).

### 2.3 Model construction and treatment

Sixty male SPF grade male Sprague-Dawley rats (170–200 g) were purchased from the Department of Medical Sciences of Peking University [Department of Experimental Animal Science, licence number SCXK (Beijing) 2017-0005]. All animals were maintained under a 12 h light/dark cycle at a constant temperature and humidity. Each animal had free access to food and water. The experimental procedure was approved by the Medical Ethics Committee of Baotou Medical College, Inner Mongolia University of Science and Technology (20170317).

A mature, stable, and reproducible one-time tracheal instillation of bleomycin was used to prepare a rat PF model in this experiment (Zhang et al., 2016b). The rats are slowly administered bleomycin through a space between the tracheal cartilage rings into the trachea and lungs to establish a PF model. Sixty rats were randomly divided into six groups according to their body weight: normal control (CON) group, model control (MOD) group, positive control prednisone acetate (PAY) groups, the high (OIL-H), medium (OIL-M) and low (OIL-L) dose *A. mongolica* oil groups. The CON group and the MOD group were treated with normal saline at a dose of 4 ml/kg daily. The dosage of PAY was 1.5 mg/kg daily, and that of OIL-H, OIL-M and OIL-L were 2.5, 5, and 7.5 ml/kg daily, respectively (Zheng et al., 2018). After 28 days, the rats were anesthetized with 3% sodium pentobarbital *via* intraperitoneal injection after the last administration to 24 h. The lung tissues were taken out for biochemical indexes and histopathological analysis. Blood samples were collected from abdominal aorta and processed for serum extraction. Serum samples were stored at −80°C until further metabolomics research. The cecums of the rats were removed immediately, and the contents were collected and stored in a refrigerator at −80°C for DNA extraction for intestinal flora research.

### 2.4 Determination of biochemical indexes and pathological analysis

The COL-III, COL-IV, HA, LN, IL-6, and IL-1β contents in the tissue homogenates were determined by the enzyme-linked immunosorbent assay. Paraffin-embedded lung tissue was cut into 3–4 μm-thick sections and stained with HE and Masson to observe pathological changes.

### 2.5 Metabolomics analysis

#### 2.5.1 LC-MS/MS acquisitions

All samples were acquired by the LC-MS system followed machine orders. Firstly, all chromatographic separations were performed using an ultra performance liquid chromatography (UPLC) system (SCIEX, United Kingdom). An ACQUITY UPLC T3 column (100 mm*2.1 mm, 1.8 µm, Waters, United Kingdom) was used for the reversed phase separation. The mobile phase consisted of solvent A (water, 0.1% formic acid) and solvent B (Acetonitrile, 0.1% formic acid). Gradient elution conditions were set as follows: 0–0.5 min, 5% B; 0.5–7 min, 5%–100% B; 7–8 min, 100% B; 8–8.1 min, 100%–5% B; 8.1–10 min, 5% B. The injection volume for each sample was 4 µL. The column oven was maintained at 35°C and the flow rate was 0.4 ml/min.

A high-resolution tandem mass spectrometer TripleTOF5600plus (SCIEX, United Kingdom) was used to detect metabolites eluted form the column. The Q-TOF operates in positive and negative ion mode respectively. The curtain gas was set 30 PSI, lon source gas1 and gas2 were set 60 PSI and an interface heater temperature was 650°C. For positive and negative ion mode, the lonspray floating voltage were set at 5000 V and 4500 V, respectively. The mass spectrometry data were acquired using the IDA mode. The TOF mass ranged from 60 to 1200 Da. The survey scans were acquired at 150 ms and total cycle time was fixed to 0.56 s. Pulser frequency value of 11 kHz and dynamic exclusion was set for 4 s. The particle signals from each scan are recorded four times in four channels and then combined and converted into data. Furthermore, in order to evaluate the stability of the LC-MS during the whole acquisition, a quality control sample (Pool of all samples) was acquired after every 10 samples.

#### 2.5.2 Data analysis of metabolomics

The Proteowizard’s MSConvert software was used to convert the raw mass spectrometer data into readable data format mzXML. The XCMS software is used for peak extraction and quality control, and CAMERA was used for additive ion annotation of the extracted substances. The MetaX software was used to identify metabolites using primary spectrometry information for database matching identification. Candidate identified substances were matched against HMDB, KEGG, and other databases for metabolite annotation to explain the physicochemical properties and biological functions of the selected metabolites. The MetaX software was used to quantify metabolites and screen differential metabolites. QC data were used for internal normalization in our study, and metabolic features with a QC relative standard deviation (RSD) > 30% were removed. The principal component analysis (PCA) and partial least squares discriminant analysis (PLS-DA) analyses were used to screen different substances for each group of serum samples. In this experiment, metabolites with variable importance in the projection (VIP) > 1 and *p* < 0.05 were screened as candidate differential metabolites for the anti-pulmonary fibrosis effect of *A. mongolica* oil. The MetaboAnalyst software 5.0 was used to analyze the metabolic pathway MetPA (http://metpa.metabolomics.ca), the pathways with a metabolic pathway impact value >0.02 and metabolic pathway significance level −Log (*P*) > 2 were identified as the key metabolic pathways affecting PF. Based on the differential metabolites involved in these key metabolic pathways, key biomarkers of PF were also identified. Observation of key biomarker changes after *A. mongolica* oil administration intervention.

### 2.6.16S rDNA high throughput sequencing

#### 2.6.1 DNA extraction

DNA from different samples of cecums contents was extracted using the E. Z.N.A.®Stool DNA Kit according to manufacturer’s instructions. The total DNA was eluted in 50 μl of Elution buffer and stored at −80°C until further PCR assessment by LC-Bio Technology Co., Ltd., Hang Zhou, Zhejiang Province, China.

#### 2.6.2 PCR amplification and 16S rDNA sequencing

The 5′ ends of the primers were tagged with specific barcodes per sample and sequencing universal primers. The primers of 515F (5′-GTGYCAGCMGCCGCGGTAA-3′), 806R (5′-GGACTACHVGGGTWTCTAAT-3′) are used. PCR amplification was performed in a total volume of 25 μl reaction mixture containing 25 ng of template DNA, 12.5 μl PCR Premix and 2.5 μl of each primer. The PCR conditions to amplify the prokaryotic 16S fragments consisted of an initial denaturation at 98°C (30 s), 32 cycles of denaturation at 98°C (10 s), annealing at 54°C (30 s), extension at 72°C (45 s) and final extension at 72°C (10 min). The PCR products were confirmed with 2% agarose gel electrophoresis. The PCR products were purified and quantified using AMPure XT beads and Qubit, separately. The size and quantity of the amplicon library were assessed with an Agilent 2100 Bioanalyzer and with the Library Quantification Kit for Illumina. The libraries were sequenced on NovaSeq PE250 platform.

#### 2.6.3 Data analysis of intestinal flora

Quality filtering of the raw reads using fqtrim (v0.94) allowed us to obtain high-quality clean tags. Chimeric sequences were filtered using the Vsearch software (v2.3.4). After dereplication using DADA2, we obtained a feature table and feature sequence. Alpha diversity and beta diversity were calculated by normalizing the data to the same sequences randomly. Alpha and beta diversity was obtained using QIIME2. Analysis of gut microbial composition and differences between groups was performed to screen for dominant flora.

### 2.7 Statistical analysis

The software SPSS19.0 was used to analyze the experimental data and the experimental results are expressed as means ± SD. The differences between all groups were analyzed using one-way analysis of variance (ANOVA). Variations between groups were considered to be significant if *p* values less than or equal to 0.05.

## 3 Results

### 3.1 Determination of biochemical indicators and histopathological analysis

**FIGURE 1:**
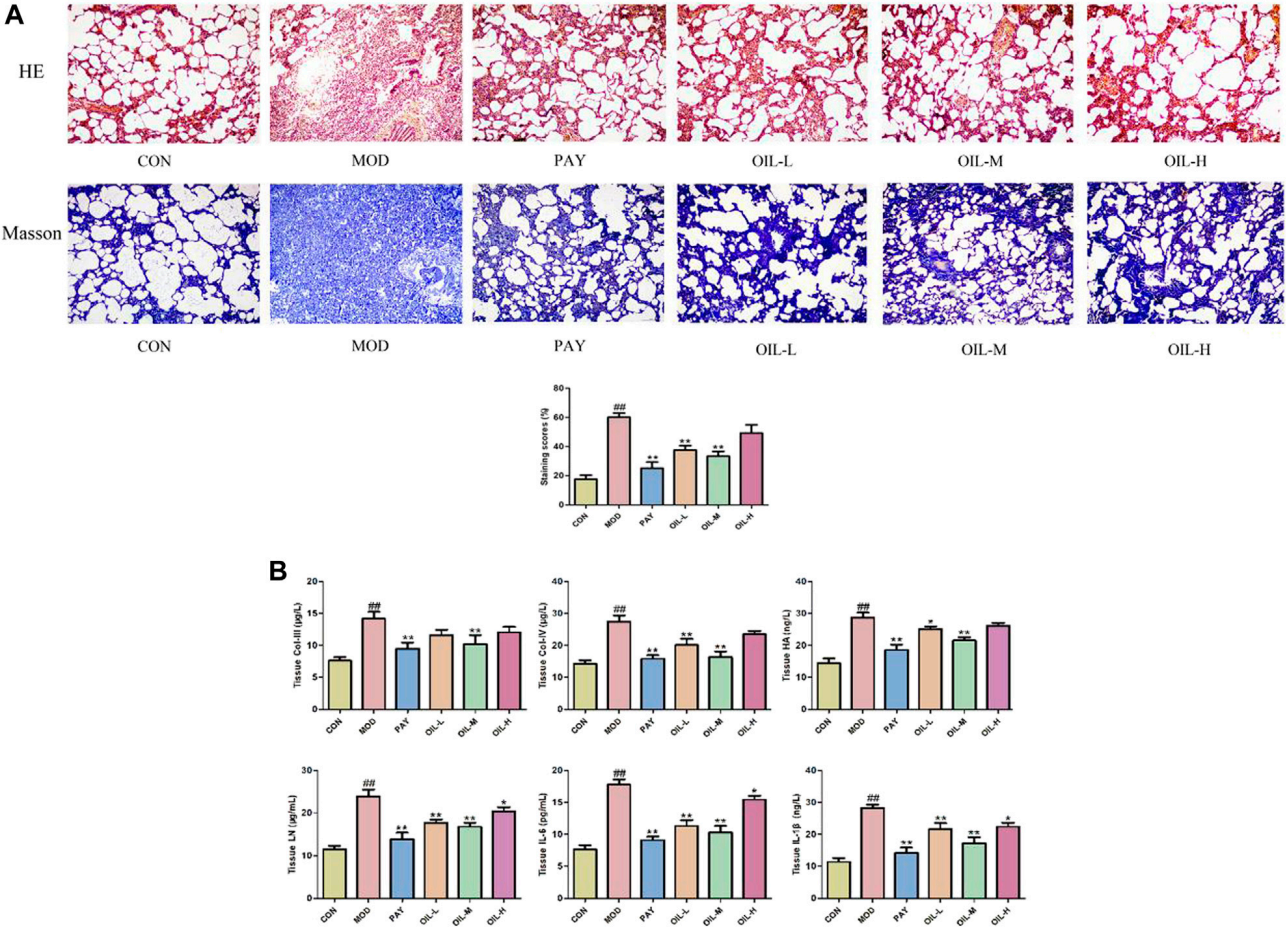
The effect of *A. mongolica* oil on histopathology of pulmonary fibrosis rats (HE, Masson×100) **(A)**. Effects of *A. mongolica* oil on tissue biochemical indices in rats with pulmonary fibrosis **(B)**. **p < 0.01,*p < 0.05 vs. model group; ##*p* < 0.01, #*p* < 0.05 vs. control group.

As shown in Figure 1A, the histopathological scores used to assess the severity of pulmonary fibrosis increased significantly in the MOD group and decreased significantly after *A. mongolica* oil administration. HE and Masson staining showed that the lung tissue structure of CON group was clear and normal. Compared with the CON group, the alveolar structure of the MOD group disappeared, interstitial edema of lung tissue, obvious infiltration of inflammatory cells, serious collagen deposition and obvious fibrosis appeared. Compared with the MOD group, the alveolar wall of rats in PAY group, OIL-L, OIL-M, and OIL-H groups became thinner, a small amount of inflammatory cell infiltration and pulmonary interstitial collagen deposition were observed, and interstitial edema was reduced. Among them, the pulmonary interstitial collagen deposition and fibrosis degree in OIL-L and OIL-M groups were significantly reduced, the alveolar structure was relatively complete, and the area of pulmonary consolidation was small.

As shown in Figure 1B, Compared with the CON group, All indexes of lung tissue in MOD group were significantly increased (*p* < 0.01). Compared with the MOD group, the contents of all biochemical indexes in lung tissue of PAY group were significantly decreased (*p* < 0.01); The contents of COL-Ⅳ, LN, IL-6, and IL-1β in lung tissue of rats in OIL-L group were significantly decreased (*p* < 0.01), and the content of HA decreased (*p* < 0.05); The contents of all biochemical indexes in lung tissue of rats in the OIL-M group decreased significantly (*p* < 0.01); The contents of LN, IL-6, and IL-1β in lung tissue of rats with OIL-H decreased significantly (*p* < 0.05).

### 3.2 Metabolomics analysis

#### 3.2.1 Screening for differential metabolites

**FIGURE 2:**
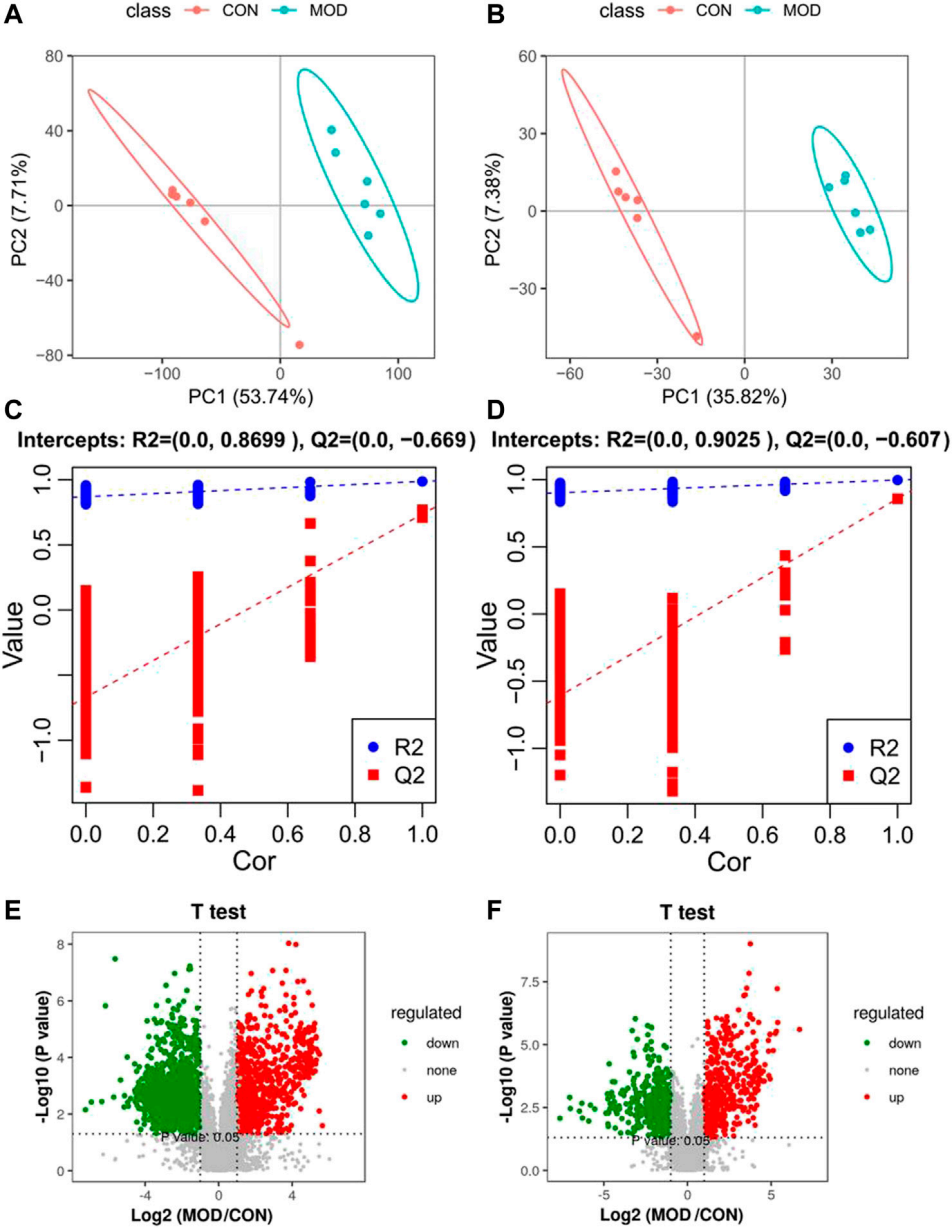
PLS-DA score diagram **(A,B)**, corresponding model verification diagram **(C,D)** and V-plot score chart **(E,F)** of control group and model group in positive and negative ion mode.

According to biochemical indexes and histopathological results, the medium dose of Mongolian almond oil was selected for metabonomic study. The quality control curves in the total ion flow chromatogram of the quality control samples in positive and negative ion modes were basically overlapped, indicating good stability of the detection system. PLS-DA was used as a supervised multivariate statistic to maximize the separation of samples. PLS-DA analysis of the CON and MOD groups in positive and negative ion mode showed significant separation and suggested that there is variability between these two groups of metabolites (Figures 2A,B). The PLS-DA models were verified using the replacement test diagram as shown in Figures 2C,D, which showed that *R* ^2^ and Q^2^ were lower than the original value on the far right from left to right and Q^2^ intersected with *Y* axis in the negative half axis, indicating good fitting and reliable results. A total of 42 putative identifications were obtained by screening and identifying with VIP >1 and *p* < 0.05 according to the Volcano Plot (V-plot) (Figures 2E,F and Table 1).

**TABLE 1:** Differential metabolites and their identification results of serum from the control and model groups.

NO.	Ratio	VIP	Common name	Chemical formula	MS1hmdb ID	Trend (M/C)
			MOD vs. CON—Positive ion mode			
1	0.17	2.05	Tetrahydrobiopterin	C_9_H_15_N_5_O_3_	HMDB0000027	↓
2	0.10	2.09	7-Hydroxy-6-methyl-8-ribit yllumazine	C_12_H_16_N_4_O_7_	HMDB0004256	↓
3	29.89	2.50	6-Hydroxy-5-methoxyindole glucuronide	C_15_H_17_NO_8_	HMDB0010362	↑
4	0.06	2.17	LysoPC [16:1 (9Z)]	C_24_H_48_NO_7_P	HMDB0010383	↓
5	0.04	2.31	LysoPC (22:0)	C_30_H_62_NO_7_P	HMDB0010398	↓
6	26.23	2.42	PE [14:0/20:5 (5Z,8Z,11Z,14Z,17Z)]	C_39_H_68_NO_8_P	HMDB0008840	↑
7	4.67	2.01	PE [O-16:1 (1Z)/22:6 (4Z,7Z,10Z,13Z,16Z,19Z)]	C_43_H_74_NO_7_P	HMDB0005780	↑
8	0.32	1.39	Ganglioside GA2 (d18:1/12:0)	C_50_H_92_N_2_O_18_	HMDB0004888	↓
9	0.46	1.08	L-Serine	C_3_H_7_NO_3_	HMDB0000187	↓
10	2.19	1.08	1-Pyrroline-5-carboxylic acid	C_5_H_7_NO_2_	HMDB0001301	↑
11	0.59	1.06	L-Histidine	C_6_H_9_N_3_O_2_	HMDB0000177	↓
12	0.37	1.09	Phenylacetylglycine	C_10_H_11_NO_3_	HMDB0000821	↓
13	0.49	1.22	Citrulline	C_6_H_13_N_3_O_3_	HMDB0000904	↓
14	3.12	1.09	4-(2-Aminophenyl)-2,4-dioxobutanoic acid	C_10_H_9_NO_4_	HMDB0000978	↑
15	3.40	1.22	Homo-L-arginine	C_7_H_16_N_4_O_2_	HMDB0000670	↑
16	6.56	1.71	Acetyl-N-formyl-5-methox ykynurenamine	C_13_H_16_N_2_O_4_	HMDB0004259	↑
17	0.17	1.56	Estradiol	C_18_H_24_O_2_	HMDB0000151	↓
18	0.22	1.03	5′-Methylthioadenosine	C_11_H_15_N_5_O_3_S	HMDB0001173	↓
19	2.89	1.12	9,12,13-TriHOME	C_18_H_34_O_5_	HMDB0004708	↑
20	2.73	1.34	N-Acetyl-b-glucosaminyla mine	C_8_H_16_N_2_O_5_	HMDB0001104	↑
21	0.37	1.16	LysoPA (0:0/16:0)	C_19_H_39_O_7_P	HMDB0007849	↓
22	4.49	1.84	Palmitoyl glucuronide	C_22_H_42_O_7_	HMDB0010331	↑
23	2.55	1.00	Deoxycytidine	C_9_H_13_N_3_O_4_	HMDB0000014	↑
24	0.42	1.23	LysoPC (15:0)	C_23_H_48_NO_7_P	HMDB0010381	↓
25	0.41	1.17	LysoPC (14:0)	C_22_H_46_NO_7_P	HMDB0010379	↓
26	0.31	1.18	LysoPC [18:3 (6Z,9Z,12Z)]	C_26_H_48_NO_7_P	HMDB0010387	↓
27	0.25	1.45	LysoPC(20:5 (5Z,8Z,11Z,14Z,17Z))	C_28_H_48_NO_7_P	HMDB0010397	↓
28	0.16	1.78	LysoPC(20:3 (5Z,8Z,11Z))	C_28_H_52_NO_7_P	HMDB0010393	↓
29	0.34	1.40	LysoPC(22:5 (4Z,7Z,10Z,13Z,16Z))	C_30_H_52_NO_7_P	HMDB0010402	↓
30	0.11	1.72	LysoPC(22:6 (4Z,7Z,10Z,13Z,16Z,19Z))	C_30_H_50_NO_7_P	HMDB0010404	↓
31	4.77	1.88	PC(15:0/18:4 (6Z,9Z,12Z,15Z))	C_41_H_74_NO_8_P	HMDB0007943	↑
32	0.20	1.46	Dimethylglycine	C_4_H_9_NO_2_	HMDB0000092	↓
33	6.41	1.42	PC(20:3 (5Z,8Z,11Z)/24:1 (15Z))	C_52_H_96_NO_8_P	HMDB0008389	↑
			MOD vs. CON—Negative ion mode			
1	0.09	2.81	Hippuric acid	C_9_H_9_NO_3_	HMDB0000714	↓
2	0.22	2.53	Indolelactic acid	C_11_H_11_NO_3_	HMDB0000671	↓
3	3.58	2.54	Indoxyl glucuronide	C_14_H_15_NO_7_	HMDB0010319	↑
4	7.79	2.69	Cytidine monophosphate	C_9_H_14_N_3_O_8_P	HMDB0000095	↑
5	0.09	2.59	3a,7a,12b-Trihydroxy-5b-c holanoic acid	C_24_H_40_O_6_	HMDB0000312	↓
6	5.99	2.47	SM (d18:0/14:0)	C_37_H_77_N_2_O_6_P	HMDB0012085	↑
7	3.32	2.21	Beta-Alanyl-CoA	C_24_H_41_N_8_O_17_P_3_S	HMDB0006805	↑
8	0.30	1.70	Homocitrulline	C_7_H_15_N_3_O_3_	HMDB0000679	↓
9	0.25	1.59	Deoxycholic acid glycine conjugate	C_26_H_43_NO_5_	HMDB0000631	↓

#### 3.2.2 Analysis of serum metabolic profile under *A. mongolica* oil intervention

**FIGURE 3:**
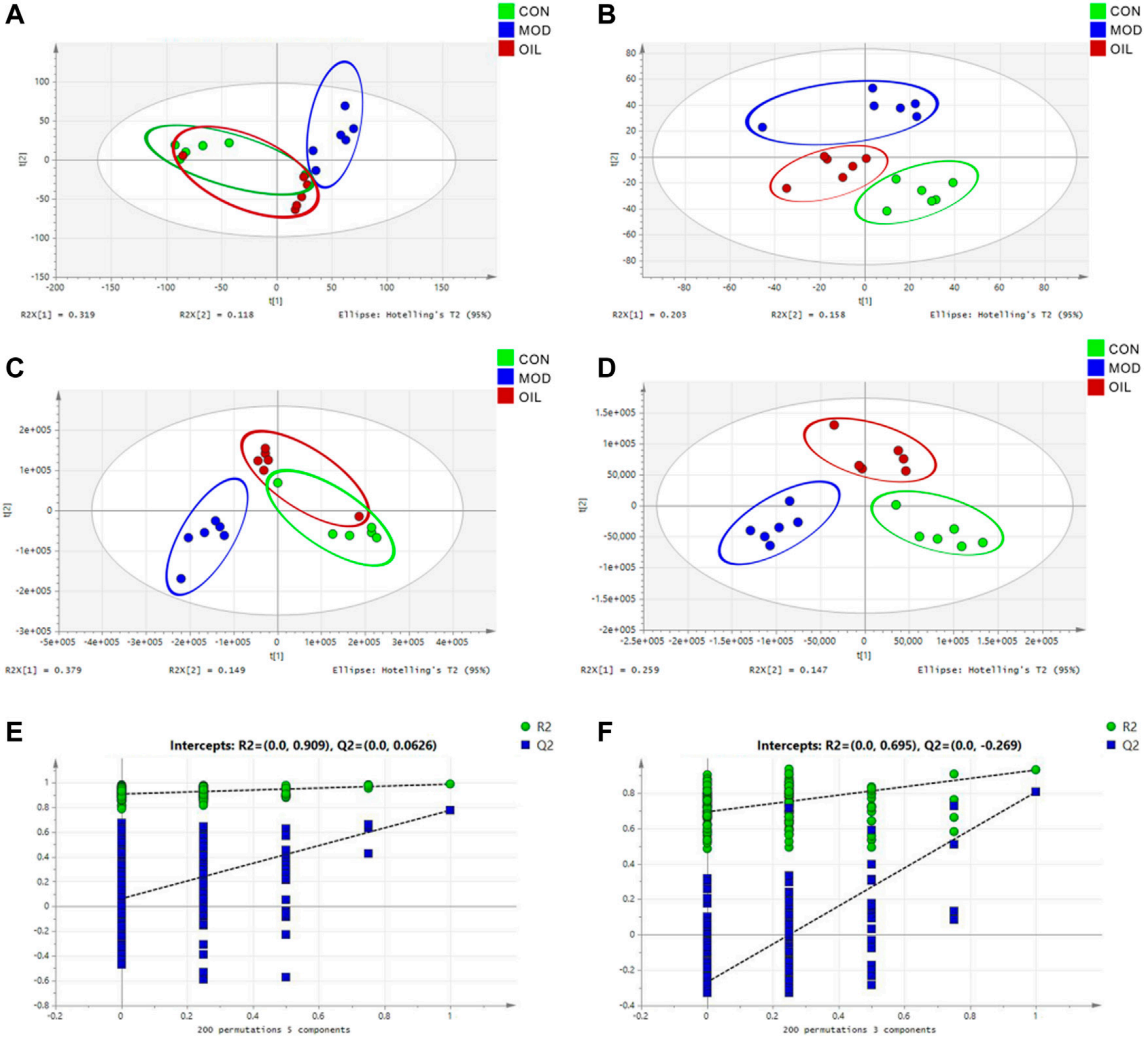
PCA score plot **(A,B)**, PLS-DA score plot **(C,D)** and validation plot of the PLS-DA model **(E,F)** of control group, model group and *A. mongolica* oil group in positive and negative ion mode.

The PCA results (Figures 3A,B) of the serum metabolic profile of rats in each group showed significant separation between the MOD and CON groups in both positive and negative ion modes. All metabolites separated and clustered well. Moreover, there was a good degree of separation between the *A. mongolica* oil group (OIL) and the MOD group, with OIL group eventually returned to the CON group. This indicates that *A. mongolica* oil has a positive regulatory effect on the endogenous metabolites of PF rats. In order to better distinguish the influence of each group on the metabolic profile, the PLS-DA is used to analyze the metabolic profile to the maximum extent (Figures 3C,D). The results showed that the metabolites of the model and the CON groups were significantly different. The OIL group was further separated from the MOD group and trended towards the CON group. The PLS-DA models were verified using class permutation tests as shown in Figures 3E,F, indicating that these models are of good fit. The predictive *R* ^2^ and Q^2^ values were 0.91 and 0.06 in the positive mode and 0.70 and 0.27 in the negative mode, respectively.

#### 3.2.3 Effects of *A. mongolica* oil on differential metabolites

**FIGURE 4:**
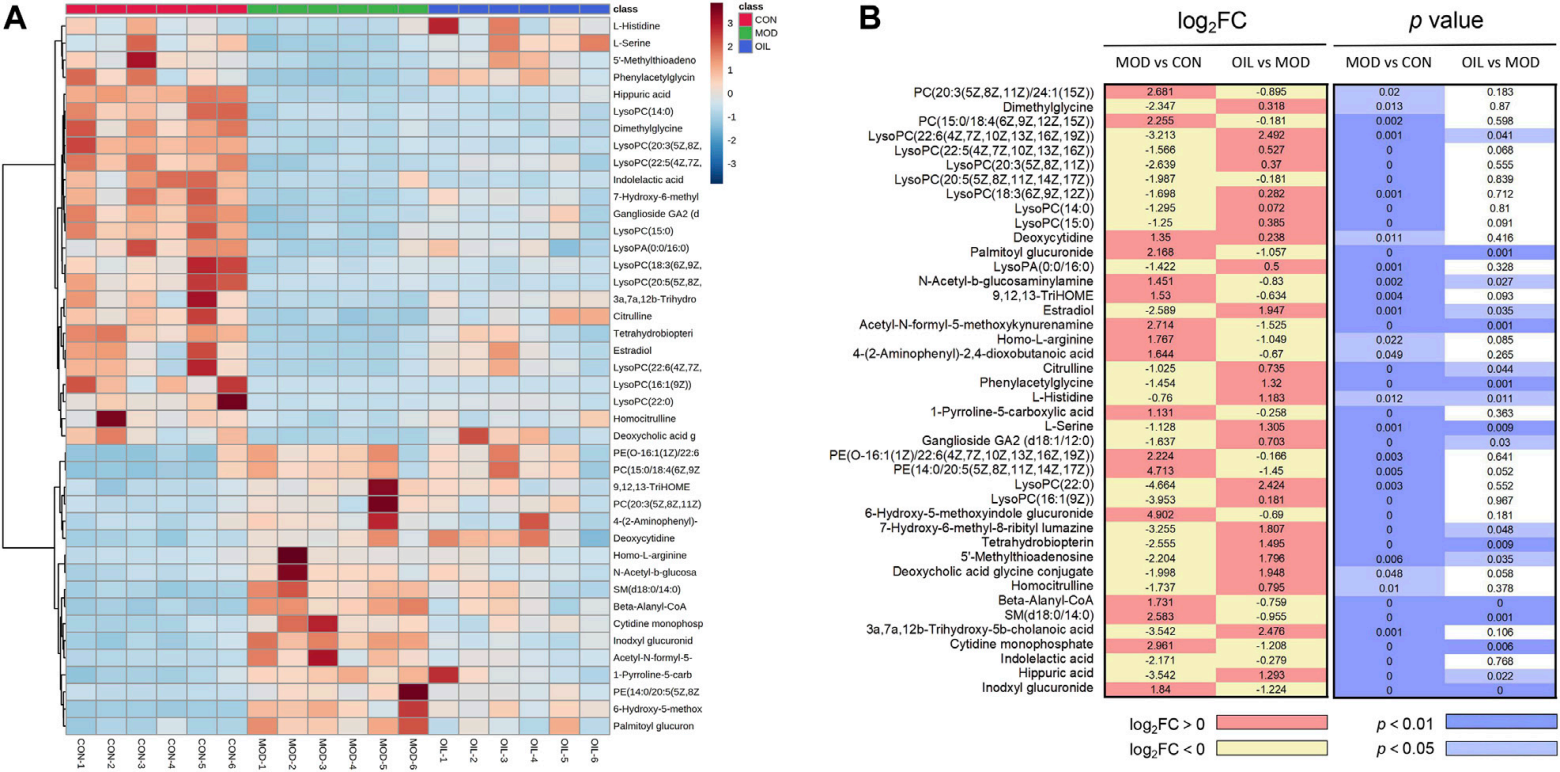
Heat map of the differentially abundant metabolites in all groups **(A)**. The degree of colour saturation indicates the metabolite expression with blue and red respectively indicating the lowest and highest expression. *p*-Value heat map of the differential abundance of metabolites in all groups **(B)**. The degree of colour saturation indicates intergroup differences in metabolite expression values with white and blue indicating non-significant and significant differences, and pink and yellow indicating upregulation and downregulation, respectively.

In order to explore the influence of *A. mongolica* oil on 42 putative identifications, a heat map cluster analysis was performed on the change trend of each group of differential metabolites, as shown in Figure 4A. The metabolites expression in model group was significantly changed compared with the normal group, and the OIL group could callback the levels of these differential metabolites which were altered by the MOD group. The *p* value heat map (Figure 4B) shows a significant difference between the model and the CON groups, as well as between the OIL group and the MOD group. Our analysis showed that compared with CON group, a total of 17 differential metabolites were significantly up-regulated and 25 were significantly down-regulated in MOD group, while seven of them were down-regulated and 11 were up-regulated in OIL group (*p* < 0.05).

#### 3.2.4 Effect of *A. mongolica* oil on metabolic pathways and key biomarkers

**FIGURE 5:**
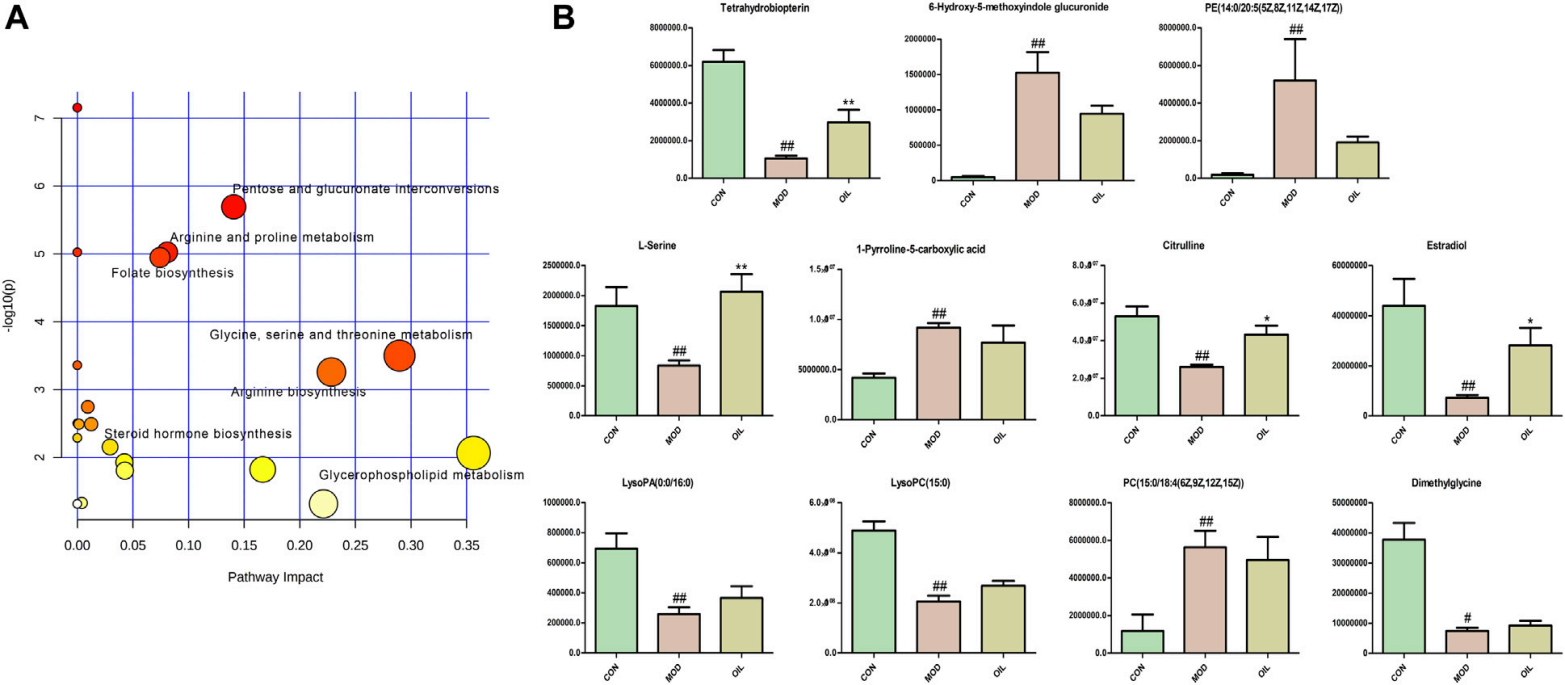
Metabolic pathway analysis of crucial biomarkers **(A)**. The size and colour of each circle indicate the pathway impact value and *p*-value respectively. Statistical significance of the 11 potential biomarkers were calculated using ANOVA after administration of *A. mongolica* oil **(B)**. *p < 0.05 and **p < 0.01 compared to the model control group. #*p* < 0.05 and ##*p* < 0.01 compared to the control group.

The 42 different metabolites initially identified in the serum of the model and CON groups were analyzed using the MetPA metabolic pathway. A total of seven key differential metabolic pathways were selected based on -Log (*P*) > 2 and Impact value >0.02 (Figure 5A): pentose and glucuronate interconversions, arginine and proline metabolism, folate biosynthesis, glycine, serine and threonine metabolism, arginine biosynthesis, steroid hormone biosynthesis and glycerophospholipid metabolism. The screening of corresponding metabolites in the pathways are the key potential biomarkers affecting PF in Table 2. The changes in key biomarkers content after administration of *A. mongolica* oil were shown in Figure 5B. Compared to the MOD group, *A. mongolica* oil significantly regulated the levels of tetrahydrobiopterin, L-serine, citrulline and estradiol based on ANOVA analysis (*p* < 0.05).

**TABLE 2:** Results of metabolic pathway analysis.

No.	Pathway name	Hits	−log(*p*)	Impact	Metabolites
1	Pentose and glucuronate interconversions	1	5.69	0.14	6-Hydroxy-5-methoxyindole glucuronide
2	Arginine and proline metabolism	1	5.02	0.08	1-Pyrroline-5-carboxylic acid
3	Folate biosynthesis	1	4.95	0.07	Tetrahydrobiopterin
4	*Glycine*, serine and threonine metabolism	2	3.50	0.29	Dimethylglycine; L-Serine
5	Arginine biosynthesis	1	3.26	0.23	Citrulline
6	Steroid hormone biosynthesis	1	2.15	0.03	Estradiol
7	Glycerophospholipid metabolism	4	2.07	0.36	LysoPA (0:0/16:0); PC[15:0/18:4 (6Z,9Z,12Z,15Z)]; PE [14:0/20:5 (5Z,8Z,11Z,14Z,17Z)]; LysoPC(15:0)

### 3.3 Analysis of intestinal flora

#### 3.3.1 Sequence diversity evaluation

**FIGURE 6:**
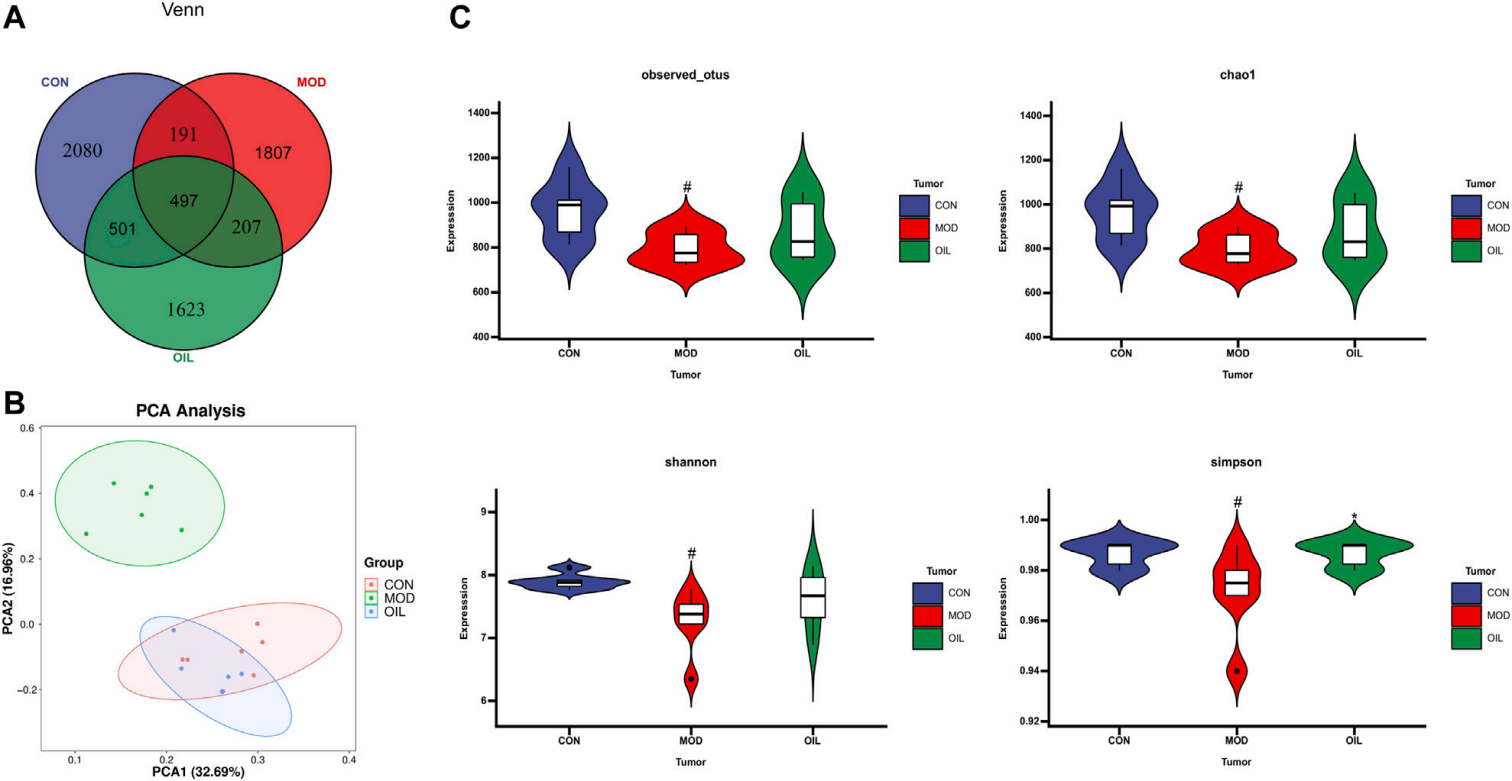
Venn diagram depicting the distribution of OTUs among different groups **(A)**. PCA score plot of control group, model group and Amygdalus mongolica oil group **(B)**. Intestinal microbial Alpha diversity of Chao1, Simpson, Shannon and Observed species in all groups **(C)**. Statistical significance was calculated with ANOVA. *p < 0.05 and **p < 0.01 compared to the model control group. #*p* < 0.05 and ##*p* < 0.01 compared to the control group.

The Venn diagram in Figure 6A shows that the CON and the MOD groups jointly detected 688 common OTU numbers. The number of OTUs shared by the OIL group and the CON group is more than the number of OTUs shared by the MOD group and CON group. It shows that the OTU of the OIL and the CON group are highly similar. PCA was used to analyze the difference of intestinal flora in each group. Figure 6B shows a significant separation between the MOD group and the CON group, indicating that there was a remarkable difference in microbial composition. The OIL group was significantly separated from the MOD group. However, the OIL group partially overlapped with the CON group, indicating that the microbial composition and structure between the samples were more similar and the difference was small. The evaluation of flora diversity mainly reflects the abundance and uniformity through Chao1, Observed species, shannon, Simpson and other indexes. The abundance and uniformity indices of the MOD group were lower than those of the CON group. But these indices increased upon treatment with *A. mongolica* oil, and simpson index was significant. (Figure 6C, *p* < 0.05).

#### 3.3.2 Analysis of intestinal microbe composition

**FIGURE 7:**
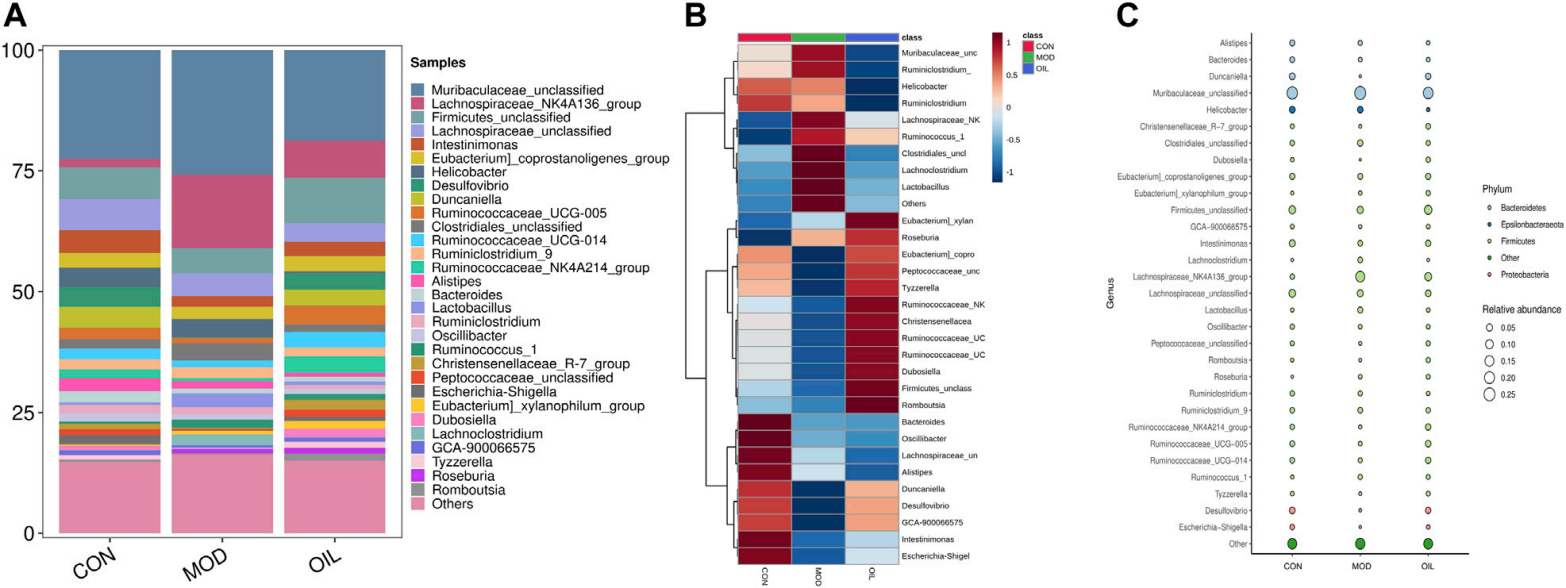
Species composition abundance map at the genus level **(A)**. Abundance clustering heat map of the genera **(B)**. The closer to blue, the lower the abundance, and the closer to red, the higher the abundance. Buble plot at the genus level **(C)**. The color represents the phylum respectively.

The highest abundance was selected to form the dominant intestinal flora at the genus level as shown in Figure 7A. There are Muribaculaceae*_unclassified*, Lachnospiraceae*_NK4A136_group*, *Firmicutes_unclassified*, Lachnospiraceae*_unclassified*, *Intestinimonas*, *Eubacterium*]*_coprostanoligenes_group*, *Helicobacter*, *Desulfovibrio*, *Duncaniella*, Ruminococcaceae*_UCG-005* and so on. They belong to the *Firmicutes*, *Bacteroidetes*, *Proteobacteria*, *Epsilonbacteraeota* and other, srespectively (Figure 7B). Clustering according to the similarity between the root samples is presented using a heat map as shown in Figure 7C. The results show that the MOD group is significantly separated from the CON group. The OIL group is clustered close to the CON group, and has a callback effect on the altered bacterial genera in the MOD group.

#### 3.3.3 Analysis of differences between groups

**FIGURE 8:**
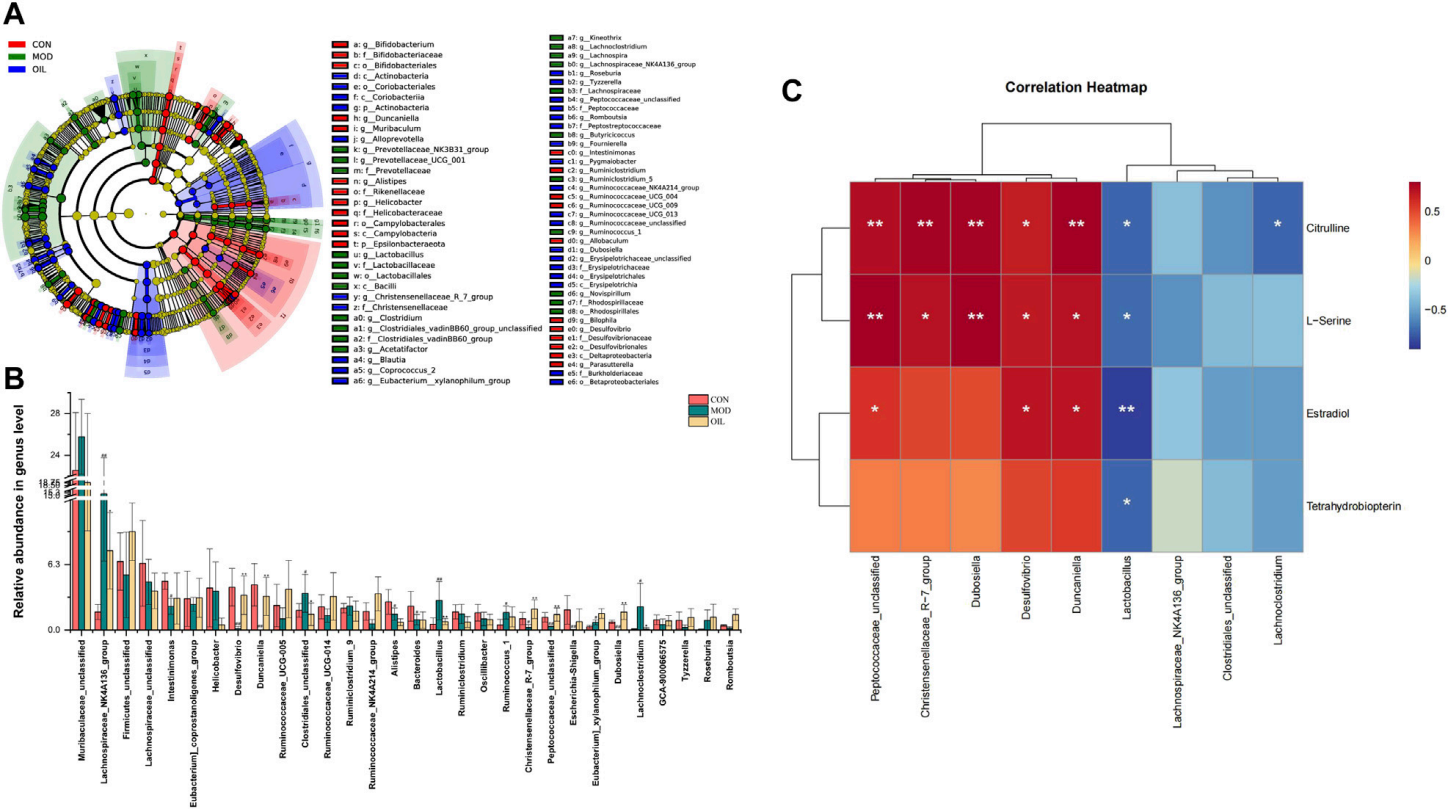
Intestinal microbial LEfSe from domain to species **(A)**. Statistical significance of the intestinal contents were calculated using ANOVA after administration of *A. mongolica* oil at the genus level **(B)**. *p < 0.05 and **p < 0.01 compared to the model control group. #*p* < 0.05 and ##*p* < 0.01 compared to the control group. Spearman analysis of potential biomarkers and key difference gut microbes **(C)**.

To further explore differences in the gut microbiota among the CON group, the MOD group and OIL group, we used LDA Effect Size (LEfSe) to recognize the specific altered bacterial phenotypes at each phylogenetic level. As Figure 8A shows that Screened the rank sum test *p* < 0.05 and LDA >3.0 were the biomarkers that were statistically different. At the genus level, the following microbiota was the most abundant in the *A. mongolica* oil: Christensenellaceae*_R-7_group*, *Eubacterium*]*_xylanophilum_group*, Peptococcaceae*_unclassified*, *Dubosiella*, Ruminococcaceae_NK4A214_group and so on*.* The changes in the microbiota among the three groups were also investigated using the ANOVA at genus level as shown in Figure 8B. Compared with the CON group, the abundance of Lachnospiraceae*_NK4A136_group*, *Clostridiales_unclassified*, *Lactobacillus*, *Ruminococcus_1*, *Eubacterium*]*_xylanophilum_group* and *Lachnoclostridium* in the MOD group were significantly increased (*p* < 0.05). On the other hand, the abundance of *Intestinimonas*, *Desulfovibrio*, *Duncaniella*, *Alistipes*, *Bacteroides*, Christensenellaceae*_R-7_group*, *Escherichia-Shigella* and *Dubosiella* were significantly reduced (*p* < 0.05). However, the abundance of Lachnospiraceae*_NK4A136_group*, *Desulfovibrio*, *Duncaniella*, *Clostridiales_unclassified*, *Lactobacillus*, Christensenellaceae*_R-7_group*, Peptococcaceae*_unclassified*, *Dubosiella* and *Lachnoclostridium* were close to the CON group significantly after the administration of *A. mongolica* oil (*p* < 0.05).

### 3.4 Correlation analysis

Correlation clustering was used to analyze the interaction between serum metabolites and intestinal flora. Spearman correlation was used to calculate the differences about the four key metabolites and nine key gut microbes which varied significantly were found in the callback to the CON group after the administration of *A. mongolica* oil. As Figure 8C shows that we can see significant positive and negative associations between identified metabolites and gut microbiota were shown as a correlation work.

## 4 Discussion

In this study, untargeted LC-MS/MS metabolomics and 16S rDNA sequencing technology were used to determine the effect of *A. mongolica* oil treatment on the intestinal flora and serum metabolite profiles of bleomycin-induced PF rat models. The results indicated for the serum metabolite profiles and intestinal flora species of rats were affected by *A. mongolica* oil treatment. Rats in the MOD group were first screened and a total 11 potential key serum biomarkers were identified: 6-Hydroxy-5-methoxyindole glucuronide, 1-Pyrroline-5-carboxylic acid, Tetrahydrobiopterin, Dimethylglycine, L-Serine, Citrulline, Estradiol, LysoPA (0:0/16:0), PC(15:0/18:4 (6Z,9Z,12Z,15Z)), PE (14:0/20:5 (5Z,8Z,11Z,14Z,17Z)) and LysoPC(15:0). These metabolites participated in Pentose and glucuronate interconversions, Arginine and proline metabolism, Folate biosynthesis, *Glycine*, serine and threonine metabolism, Arginine biosynthesis, Steroid hormone biosynthesis and Glycerophospholipid metabolism. Tetrahydrobiopterin, L-Serine, Citrull *A. mongolica* and Estradiol demonstrated significant callback compared to controls after oil treatment. And the abundance of Lachnospiraceae*_NK4A136_group*, *Desulfovibrio*, *Duncaniella*, *Clostridiales_unclassified*, *Lactobacillus*, Christensenellaceae*_R-7_group*, Peptococcaceae*_unclassified*, *Dubosiella* and *Lachnoclostridium* were close to the CON group significantly after the administration of *A. mongolica* oil. The results show that *A. mongolica* oil affected the levels of PF metabolites and the abundance of certain intestinal flora through multiple targets, thereby improving PF.

L- Serine participates in the biosynthesis of macromolecules such as protein, nucleic acids, and lipids. It is considered to be the third largest metabolite after glucose and glutamine (Frezza, 2016). It is involved in pathophysiology of diabetes and neuropsychiatric diseases, regulation of tumor development, and inhibition of macrophage and neutrophil-mediated inflammatory response (Amelio et al., 2014; El-Hattab, 2016; He et al., 2019; Holm and Buschaed, 2019). As a non-protein amino acid, Citrulline takes part in peripheral protein homeostasis and is a precursor of *de novo* arginine synthesis in the kidneys, endothelial cells and immune cells (Bahri et al., 2013). Citrulline has roles in the treatment of rheumatoid arthritis, gastrointestinal function, multiple sclerosis, fibrosis, as well as several other diseases (Crenn et al., 2008; Gudmann et al., 2015; Darrah and Andrade, 2018). Citrulline is able to establish an anti-inflammatory microenvironment by increasing interleukin-10 and reducing IL-1β and interleukin-12 in human proximal tubular cells. It is also able to significantly reduce urinary albumin excretion, renal tubulointerstitial fibrosis, and kidney hypertrophy (Romero et al., 2013). The results of this study show that the *A. mongolica* oil treatment increases the level of nutritional amino acids with anti-inflammatory and antioxidant properties such as L-serine and citrulline, thereby enhancing the body’s ability to promote the expression of anti-inflammatory and antioxidant related genes to productive against PF. Estradiol is an antioxidant that can inhibit lipid peroxidation, loss of antioxidant enzyme activity and liver fibrosis (Lu et al., 2004). It reduces glomerular sclerosis and tubulointerstitial fibrosis by reducing extracellular matrix synthesis and increasing extracellular matrix degradation, thereby playing a role in renal protection (Mankhey et al., 2007). Tetrahydrobiopterin can improve the vascular endothelial function of patients with cystic fibrosis and those with advanced myocardial hypertrophy and fibrosis. It may reduce PF-induced pulmonary hypertension and pulmonary artery endothelial-mesenchymal transition (Moens et al., 2008; Almudéver et al., 2013; Jeong et al., 2019). *A. mongolica* oil enhances the levels of estradiol and tetrahydrobiopterin, resulting in reduced extracellular matrix formation in rats with PF.

**FIGURE 9:**
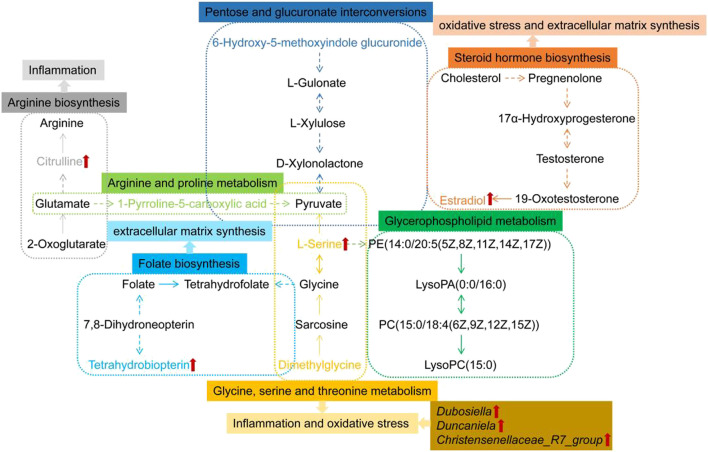
Metabolic networks of differential metabolites in response to PF and the preventive effect of *A. mongolica* oil. Key biomarkers for *A. mongolica* oil callbacks in red arrow.

Studies in humans and other mammals have revealed the effects of intestinal flora profiles on a range of physiologic processes which are affecting energy homeostasis, metabolism and immunologic activity among several other systems (Barko et al., 2017). The effect of the intestinal microbiota on the lungs is known as the “gut-lung” axis, and most of the inflammation involved in the alteration of bacteria or bacterial products crosses the gastrointestinal barrier into the blood vessels (Bingula et al., 2017). Dubosiella as beneficial bacteria, showed a decrease abundances in the PF mice model induced by irradiation of X-ray in Li’s research (Li et al., 2020). This is in accordance with our results. Additional studies demonstrated that Antrodin A from *Antrodia camphorata* can increase the relative abundance of *Dubosiella* and restore the composition of the intestinal flora to improve liver fat deposition, oxidative stress and inflammation induced by alcohol exposure and liver injury (Yi et al., 2021). Resistant dextrin can increase the relative abundance of *Dubosiella* to improved intestinal injury and reduce levels of pro-inflammatory cytokines and intestinal permeability (Zhang et al., 2021). Faecalibacterium prausnitzii can improve microbial imbalance, while higher levels of *Dubosiella* protects against allergic asthma caused by house dust mites and also works to reduce allergic airway inflammation (Hu et al., 2021b). β-globulin has anti-oxidant, antibacterial, and immunomodulatory properties. Rats fed with β-globulin showed an increased abundance of *Duncaniella* (Xia et al., 2020). Christensenellaceae*_R-7_group* may be involved in amino acid, peptide and lipid metabolism (Gong et al., 2020). Continuous probiotic supplementation increases the abundance of Christensenellaceae*_R7_group* (Khan and Chousalkar, 2020)*.* Our study demonstrates that the abundance of *Dubosiella*, *Duncaniela* and Christensenellaceae*_R7_group* were increased after *A. mongolica* oil treatment compared to the MOD group and effectively regulated intestinal microbiota while inhibiting the infiltration of inflammatory cells and lung tissue fibrosis (Figure 9).

## 5 Conclusion

Using untargeted LC-MS/MS metabolomics and 16S rDNA sequencing technology, we show that the *A. mongolica* oil can increase the level of Tetrahydrobiopterin, L-Serine, Citrulline and Estradiol to reduce oxidative and inflammatory damage, regulate the levels of extracellular matrix, resulting an overall improvement in rat models of PF. Additionally, it also can increase the abundance of *Dubosiella*, *Duncaniela*, Christensenellaceae*_R7_group* and so on to increase intestinal flora diversity and improve inflammation to improve PF. Following research should aim to verify the relationship between intestinal flora and key metabolic pathways. This study provides experimental and scientific basis supporting the protective effect of *A. mongolica* oil on fibrotic lungs, while providing new ideas for the rational development and utilization of *A. mongolica* oil resources in the pharmaceutical and food industries at the same time.

## Data Availability

The data presented in the study are deposited in the Genome Sequence Archive (Genomics, Proteomics & Bioinformatics 2021) in National Genomics Data Center (Nucleic Acids Res 2022), China National Center for Bioinformation/Beijing Institute of Genomics, Chinese Academy of Sciences (GSA: CRA008575) that are publicly accessible at https://ngdc.cncb.ac.cn/gsa.

## References

[B1] Almudéver P. Milara J. De Diego A. Serrano-Mollar A. Xaubet A. Perez-Vizcaino F. (2013). Role of tetrahydrobiopterin in pulmonary vascular remodelling associated with pulmonary fibrosis. Thorax 68 (10), 938–948. 10.1136/thoraxjnl-2013-203408 23739137

[B2] Amelio I. Markert E. K. Rufini A. Antonov A. V. Sayan B. S. Tucci P. (2014). P73 regulates serine biosynthesis in cancer. Oncogene 33 (42), 5039–5046. 10.1038/onc.2013.456 24186203

[B3] Bahri S. Zerrouk N. Aussel C. Moinard C. Crenn P. Curis E. (2013). Citrulline: From metabolism to therapeutic use. Nutrition 29 (3), 479–484. 10.1016/j.nut.2012.07.002 23022123

[B4] Bai W. F. Shi S. L. Zhou H. B. Zhang Y. (2015). Comparision of contents of amygdalin in *Amygdalus mongolica* seed from different producing area. Chin. J. Health Lab. Technol. 25, 315–317.

[B5] Barko P. C. McMichael M. A. Swanson K. S. Williams D. A. (2017). The gastrointestinal microbiome: A review. J. Vet. Intern. Med. 32 (1), 9–25. 10.1111/jvim.14875 29171095 PMC5787212

[B6] Bingula R. Filaire M. Radosevic-Robin N. Bey M. Berthon J. Y. Bernalier-Donadille A. (2017). Desired turbulence? Gut-lung Axis, immunity, and lung cancer. J. Oncol. 2017, 5035371. 10.1155/2017/5035371 29075294 PMC5623803

[B7] Chanda D. Otoupalova E. Smith S. R. Volckaert T. De Langhe S. P. Thannickal V. J. (2019). Developmental pathways in the pathogenesis of lung fibrosis. Mol. Asp. Med. 65, 56–69. 10.1016/j.mam.2018.08.004 PMC6374163 30130563

[B8] Chang H. Bai W. F. Li Q. Liu Q. L. Meng H. Y. Shi S. L. (2021). Dynamic study on protective effects of *Amygdala mongolica* on hepatic fibrosis based on changes of serum biomarkers. Chin. Tradit. Herb. Drugs. 52, 108–117.

[B9] Chang H. Liu Q. Bai W. F. Bai Y. C. Jia X. Y. Gao C. (2020). Protective effects of *Amygdalus mongolica* on rats with renal fibrosis based on serummetabolomics. J. Ethnopharmacol. 257, 112858. 10.1016/j.jep.2020.112858 32278030

[B10] Clos-Garcia M. Andrés-Marin N. Fernández-Eulate G. Abecia L. Lavín J. L. van Liempd S. (2019). Gut microbiome and serum metabolome analyses identify molecular biomarkers and altered glutamate metabolism in fibromyalgia. EBioMedicine 46, 499–511. 10.1016/j.ebiom.2019.07.031 31327695 PMC6710987

[B11] Clos-Garcia M. Garcia K. Alonso C. Iruarrizaga-Lejarreta M. D'Amato M. Crespo A. (2020). Integrative analysis of fecal metagenomics and metabolomics in colorectal cancer. Cancers (Basel) 12 (5), 1142. 10.3390/cancers12051142 PMC7281174 32370168

[B12] Crenn P. Messing B. Cynober L. (2008). Citrulline as a biomarker of intestinal failure due to enterocyte mass reduction. Clin. Nutr. 27 (3), 328–339. 10.1016/j.clnu.2008.02.005 18440672

[B13] Darrah E. Andrade F. (2018). Rheumatoid arthritis and citrullination. Curr. Opin. Rheumatol. 30 (1), 72–78. 10.1097/BOR.0000000000000452 28937414 PMC5848217

[B14] Dumas A. Bernard L. Poquet Y. Lugo-Villarino G. Neyrolles O. (2018). The role of the lung microbiota and the gut-lung axis in respiratory infectious diseases. Cell. Microbiol. 20 (12), e12966. 10.1111/cmi.12966 30329198

[B15] El-Hattab A. W. (2016). Serine biosynthesis and transport defects. Mol. Genet. Metab. 118 (3), 153–159. 10.1016/j.ymgme.2016.04.010 27161889

[B16] Frezza C. (2016). Cancer metabolism: Addicted to serine. Nat. Chem. Biol. 12 (6), 389–390. 10.1038/nchembio.2086 27191646

[B17] Gong S. Ye T. Wang M. Wang M. Li Y. Ma L. (2020). Traditional Chinese medicine formula kang shuai Lao pian improves obesity, gut dysbiosis, and fecal metabolic disorders in high-fat diet-fed mice. Front. Pharmacol. 11, 297. 10.3389/fphar.2020.00297 32269525 PMC7109517

[B18] Gosalbes M. J. Abellan J. J. Durbán A. Pérez-Cobas A. E. Latorre A. Moya A. (2012). Metagenomics of human microbiome: Beyond 16s rDNA. Clin. Microbiol. Infect. 18, 47–49. 10.1111/j.1469-0691.2012.03865.x 22647049

[B19] Gudmann N. S. Hansen N. U. Jensen A. C. Karsdal M. A. Siebuhr A. S. (2015). Biological relevance of citrullinations: Diagnostic, prognostic and therapeutic options. Autoimmunity 48 (2), 73–79. 10.3109/08916934.2014.962024 25520183

[B20] Hao H. M. Shi S. L. Zhou H. B. Liu Q. Bai W. F. Chang H. (2020). Protective effect of *Amygdalus Mongolica* oil on the kidneys in rats with renal fibrosis. Pharmacol. Clin. Chin. Mat. Med. 36, 105–109.

[B21] He F. Yin Z. Wu C. Xia Y. Wu M. Li P. (2019). l-Serine lowers the inflammatory responses during pasteurella multocida infection. Infect. Immun. 87 (12), e00677. 10.1128/IAI.00677-19 31570555 PMC6867830

[B22] He Y. Wen Q. Yao F. Xu D. Huang Y. Wang J. (2017). Gut-lung axis: The microbial contributions and clinical implications. Crit. Rev. Microbiol. 43, 81–95. 10.1080/1040841X.2016.1176988 27781554

[B23] Holm L. J. Buschaed K. (2019). L-Serine: A neglected amino acid with a potential therapeutic role in diabetes. APMIS 127 (10), 655–659. 10.1111/apm.12987 31344283 PMC6851881

[B24] Hu J. B. Li J. Yang J. S. (2021). Advances in the pathogenesis and clinical treatment of idiopathic pulmonary fibrosis. Jilin Med. J. 42 (12), 3020–3023.

[B25] Hu W. Lu W. Li L. Zhang H. Lee Y. K. Chen W. (2021). Both living and dead Faecalibacterium prausnitzii alleviate house dust mite-induced allergic asthma through the modulation of gut microbiota and short-chain fatty acid production. J. Sci. Food Agric. 101 (13), 5563–5573. 10.1002/jsfa.11207 33709404

[B26] Hu X. Xie Y. Xiao Y. Zeng W. Gong Z. Du J. (2020). Longitudinal analysis of fecal microbiome and metabolome during renal fibrotic progression in a unilateral ureteral obstruction animal model. Eur. J. Pharmacol. 886, 173555. 10.1016/j.ejphar.2020.173555 32937112

[B27] Jeong J. H. Lee N. Tucker M. A. Rodriguez-Miguelez P. Looney J. Thomas J. (2019). Tetrahydrobiopterin improves endothelial function in patients with cystic fibrosis. J. Appl. Physiol. 126 (1), 60–66. 10.1152/japplphysiol.00629.2018 30433862

[B28] Jia X. Y. Gao C. Liu Q. Bai Y. C. Shi S. L. (2018). Determination of total alkaloids in *Amygdalus mongolica* . Guangzhou Chem. Ind. 46, 100–102.

[B29] Jia X. Y. Gao C. Liu Q. Shi S. L. Zhou H. B. Bai Y. C. (2020). Screening of effective extraction sites of *Amygdalus mongolica* medicinalmaterials against rat renal fibrosis. Sci. Technol. Food Ind. 41, 297–308.

[B30] Khan S. Chousalkar K. K. (2020). Salmonella Typhimurium infection disrupts but continuous feeding of Bacillus based probiotic restores gut microbiota in infected hens. J. Anim. Sci. Biotechnol. 11, 29. 10.1186/s40104-020-0433-7 32211190 PMC7087389

[B31] Li Q. Bai W. F. Zhou H. B. Hao H. M. Li X. Chang H. (2021). Protective effect of *Amygdalus Mongolica* oil on lung in rats with pulmonary fibrosis. Pharmacol. Clin. Chin. Mate.r Med. 37 (4), 90–96.

[B32] Li W. Lu L. Liu B. Qin S. (2020). Effects of phycocyanin on pulmonary and gut microbiota in a radiation-induced pulmonary fibrosis model. Biomed. Pharmacother. 132, 110826. 10.1016/j.biopha.2020.110826 33068929 PMC7556228

[B33] Liu G. R. Li Y. Tian Y. G. (2019). Research progress of traditional Chinese medicine on pulmonary fibrosis. Chin. Medi. Mod. Dis. Educ. China. 17, 116–119.

[B34] Liu P. Liu S. Tian D. Wang P. (2012). The applications and obstacles of metabonomics in traditional Chinese medicine. Evid. Based. Complement. Altern. Med. 2012, 945824. 10.1155/2012/945824 PMC3155802 21860656

[B35] Liu X. J. Zhang Y. Q. Li J. (2017). Research of bencaological study and chemicalconstiutents of Pruni Semen. J. Liaoning Univ. Tradit. Chin. Med. 19, 100–103.

[B36] Lu G. Shimizu I. Cui X. Itonaga M. Tamaki K. Fukuno H. (2004). Antioxidant and antiapoptotic activities of idoxifene and estradiol in hepatic fibrosis in rats. Life Sci. 74 (7), 897–907. 10.1016/j.lfs.2003.08.004 14659978

[B37] Lyu S. Yang S. L. Rao Y. Feng Y. L. (2018). Application of metabolomics and related technologies in research and development field of traditional Chinese medicine. China. J. Chin. Mat. Med. 43, 4182–4191. 10.19540/j.cnki.cjcmm.20180709.005 30583615

[B38] Mankhey R. W. Wells C. C. Bhatti F. Maric C. (2007). 17beta-Estradiol supplementation reduces tubulointerstitial fibrosis by increasing MMP activity in the diabetic kidney. Am. J. Physiol. Regul. Integr. Comp. Physiol. 292 (2), 769–777. 10.1152/ajpregu.00375.2006 16931652

[B39] Martinez F. J. Collard H. R. Pardo A. Raghu G. Richeldi L. Selman M. (2017). Idiopathic pulmonary fibrosis. Nat. Rev. Dis. Prim. 3, 17074. 10.1038/nrdp.2017.74 29052582

[B40] Moens A. L. Takimoto E. Tocchetti C. G. Chakir K. Bedja D. Cormaci G. (2008). Reversal of cardiac hypertrophy and fibrosis from pressure overload by tetrahydrobiopterin: Efficacy of recoupling nitric oxide synthase as a therapeutic strategy. Circulation 117 (20), 2626–2636. 10.1161/CIRCULATIONAHA.107.737031 18474817 PMC2614930

[B41] Prasse A. Holle J. U. Müller-Quernheim J. (2010). Pulmonary fibrosis. Internist (Berl) 51 (1), 6–13. 10.1007/s00108-009-2406-y 19956919

[B42] Quan B. W. Wu T. Liu Q. Gao C. Zhou H. B. Bai Y. C. (2020). Protective effect of different polar parts of *Amygdalus mongolica* on pulmonary fibrosis rat models induced by bleomycin. Sci. Technol. Food Ind. 41, 305–309.

[B43] Romero M. J. Yao L. Sridhar S. Bhatta A. Dou H. Ramesh G. (2013). l-Citrulline protects from kidney damage in type 1 diabetic mice. Front. Immunol. 4, 480. 10.3389/fimmu.2013.00480 24400007 PMC3871963

[B44] Shi C. Lin L. L. Xie T. Shen C. S. Ji J. J. Zhao X. (2020). Exploring the influence of pulmonary and intestinal microecology on lung diseases based on the "lung-gut. axis, JNMU. 36 (02), 168–173.

[B45] Shi S. L. Bai Y. C. Zhou H. B. Niu S. F. (2013). Extraction and determination of content of polysaccharides in *Amygdalus mongolica* . Lishizhen Med. Mat. Med. Res. 24, 257–258.

[B46] Song J. P. Du B. R. Zhang R. Li S. Y. Zhou M. Fang H. (2010). Investigation on clinical manifestations in common of pulmonary fibrosis. Chin. J. Basic Med. TCM. 16, 141–142.

[B47] Su K. Shi S. L. Zheng D. H. Li J. H. (2013). Determination of alpha tocopherol content in *Amygdalus mongolica* by HPLC. Chin. J. Exp. Tradit. Med. Formulae 19, 70–72.

[B48] Tan J. Y. Tang Y. C. Huang J. (2020). Gut microbiota and lung injury. Adv. Exp. Med. Biol. 12, 55–72. 10.1007/978-981-15-2385-4_5 32323180

[B49] Wang L. M. Wang P. Teka T. Zhang Y. C. Yang W. Z. Zhang Y. (2020). 1H NMR and UHPLC/Q-Orbitrap-MS-Based metabolomics combined with 16S rRNA gut microbiota analysis revealed the potential regulation mechanism of nuciferine in hyperuricemia rats. J. Agric. Food Chem. 68 (47), 14059–14070. 10.1021/acs.jafc.0c04985 33146009

[B50] Wang Y. L. Li Y. H. W J. Zhu Q. Wang L. (2012). Analysis of nutritional components of three kind of Amygdalus splant. Guangdong agri. Sci. 39, 127–129.

[B51] Wang Q. Zhao Y. Q. Shen X. L. Xiao Q. L. (2021). Research progress in the treatment of pulmonary fibrosis in Chinese medicine. Hebei Med. 27 (10), 1751–1753.

[B52] Weir T. L. Manter D. K. Sheflin A. M. Barnett B. A. Heuberger A. L. Ryan E. P. (2013). Stool microbiome and metabolome differences between colorectal cancer patients and healthy adults. PLoS One 8 (8), e70803. 10.1371/journal.pone.0070803 23940645 PMC3735522

[B53] Wu G. S. Li H. K. Zhang W. D. (2019). Metabolomics and its application in the treatment of coronary heart disease with traditional Chinese medicine. Chin. J. Nat. Med. 17 (5), 321–330. 10.1016/S1875-5364(19)30037-8 31171266

[B54] Wu T. Zhou H. B. Wang J. Chang H. Bai W. F. Quan B. W. (2021). Effect of different solvent extracts of *Amygdalus mongolica*on liver fibrosis rat models induced by carbon tetrachloride and its mechanisms. Sci. Technol. Food Ind. 42 (14), 348–355.

[B55] Xia Y. Fukunaga M. Kuda T. Goto M. Chiaraluce G. Hoshiba H. (2020). Detection and isolation of protein susceptible indigenous bacteria affected by dietary milk-casein, albumen and soy-protein in the caecum of ICR mice. Int. J. Biol. Macromol. 144, 813–820. 10.1016/j.ijbiomac.2019.09.159 31743706

[B56] Yang M. Q. Zhou H. B. Bai Y. C. Shi S. L. (2018). Determination of flavonoids inleaves of *Amygdalus mongolica* from 6 different areas. Guangzhou Chem. Ind. 46, 77–79.

[B57] Yi Z. Liu X. Liang L. Wang G. Xiong Z. Zhang H. (2021). Antrodin A from Antrodia camphorata modulates the gut microbiome and liver metabolome in mice exposed to acute alcohol intake. Food Funct. 12 (7), 2925–2937. 10.1039/d0fo03345f 33720247

[B58] Yue X. Li J. W. Wu H. Y. Wang T. B. Si Q. G. W. Wang Z. Z. (2020). Experimental study on container seedling of *Amygdalus mongolica* as a desert native treeSpecies in WulatPlateau of lnner Mongolia. Inn. Mong. For. Inves. Des. 43, 33–36.

[B59] Zhan X. Liu B. Tong Z. H. (2020). Postinflammatroy pulmonary fibrosis of COVID-19: The current status and perspective. Chin. J. Tub. Res. Dis. 43 (9), 728–732. 10.3760/cma.j.cn112147-20200317-00359 32894907

[B60] Zhang A. Sun H. Wang X. (2012). Recent highlights of metabolomics for traditional Chinese medicine. Pharmazie 67, 667–675. 22957430

[B61] Zhang K. Si X. P. Huang J. Han J. Liang X. Xu X. B. (2016). Preventive effects of *Rhodiola rosea* L. on bleomycin-induced pulmonary fibrosis in rats. Int. J. Mol. Sci. 17 (6), 879. 10.3390/ijms17060879 PMC4926413 27271612

[B62] Zhang X. Q. Se C. B. T. Wu R. G. M. L. (2016). The measurement of the chemical characteristic constants of *prunus mongolica* oil. J. Jining Norm. Univer. 38, 13–15.

[B63] Zhang Z. Chen X. Cui B. (2021). Modulation of the fecal microbiome and metabolome by resistant dextrin ameliorates hepatic steatosis and mitochondrial abnormalities in mice. Food Funct. 12 (10), 4504–4518. 10.1039/d1fo00249j 33885128

[B64] Zhao Y. S. Zheng Q. N. Shi S. L. Liu Q. L. Su K. Zhou H. B. (2016). Study on solvent extraction and fatty acids composition of *Amygdalus mongolica* oil. Food Res. Dev. 37, 34–37.

[B65] Zhao Y. Z. (1995). Study on floristic geographical distribution of *Amygdalus mongolica* . J. Inn. Mong. Univ. Natur. Sci. Edit.) 6, 713–715.

[B66] Zheng Q. N. Feng C. M. Wu T. Zhou H. B. Shi S. L. (2018). Effect of *Amygdalus mongolica* Oil on the liver and mechanisms in hyperlipidemic rats. Sci. Technol. Food Ind. 39, 286–292.

[B67] Zheng Q. N. Wang J. Zhou H. B. Niu S. F. Liu Q. L. Yang Z. J. (2017). Effectiveness of *Amygdalus mongolica* oil in hyperlipidemic rats and underlying antioxidant processes. J. Toxicol. Environ. Health. A 80 (22), 1193–1198. 10.1080/15287394.2017.1367124 28949828

[B68] Zhou B. Yuan Y. Zhang S. Guo C. Li X. Li G. (2020). Intestinal flora and disease mutually shape the regional immune system in the intestinal tract. Front. Immunol. 11, 575. 10.3389/fimmu.2020.00575 32318067 PMC7147503

[B69] Zhou H. B. Niu S. F. Shi S. L. Wang Y. C. (2015). Determination of the contents of protein in different parts of *Amygdalus mongolica* . J. Baotou Med. Coll. 31, 3–4.

[B70] Zhu C. Hu P. Liang Q. L. Wang Y. M. Luo G. A. (2008). Integration of metabonomics technology and its application in modernization of traditional Chinese medicine. Pharm. Sin. 43 (7), 683–689. 18819470

[B71] Zhu Q. Li R. Wang Y. Li Y. H. (2013). Determination and analysis of oil fatty acid content in *Amygdalus mongolica* . North. Hortic. 17, 32–34.

