# Supplementary material for: The intervention effect of Amygdalus mongolica oil on the metabolomics and intestinal flora in pulmonary fibrosis
Source: Front Pharmacol. 2022 Nov 1;13:1037563. doi: 10.3389/fphar.2022.1037563 (PMC9663812; doi:10.3389/fphar.2022.1037563)
Supplement: Supplementary file 1 [file DataSheet1.PDF]

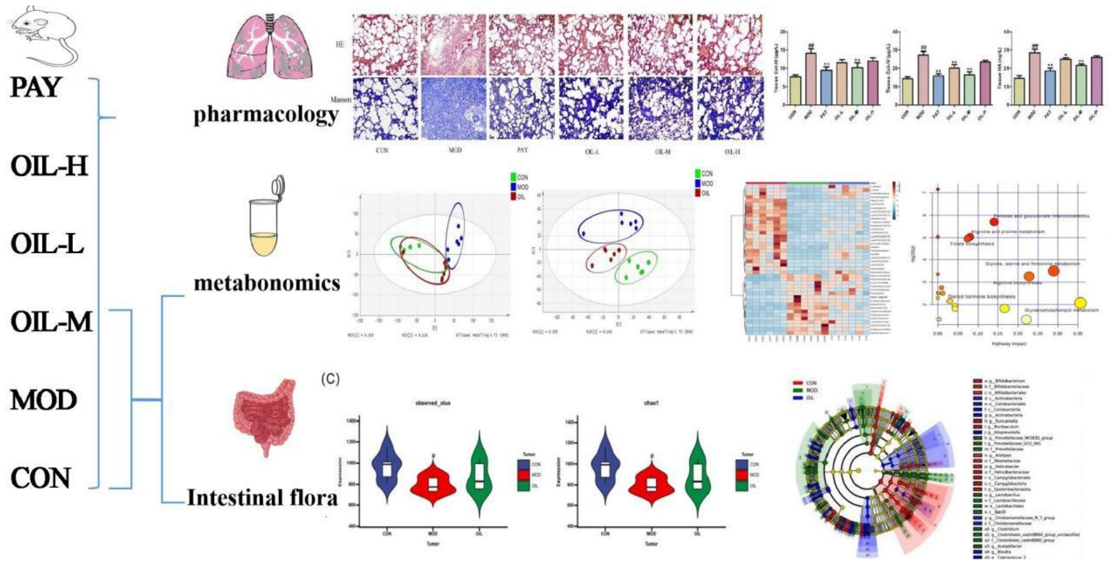

In this study, pharmacology, LC-MS/MS metabonomics and 16S rDNA sequencing technology were used to explore the effect and mechanism of *Amygdalus mongolica* oil on pulmonary fibrosis in rats.and found that *Amygdalus mongolica* oil appears to confer protective effects against pulmonary fibrosis by affecting the level of pulmonary fibrosis metabolites and the abundance of related intestinal flora through multiple targets.
